# Sprint skating profile of competitive female bandy players: An analysis of positional and playing-level differences

**DOI:** 10.3389/fphys.2023.1094170

**Published:** 2023-01-25

**Authors:** Roland van den Tillaar, Haris Pojskic, Håkan Andersson

**Affiliations:** ^1^ Department of Sports Sciences, Nord University, Levanger, Norway; ^2^ Department of Sports Science, Linnaeus University, Kalmar, Sweden; ^3^ High Performance Centre, Växjö, Sweden

**Keywords:** acceleration, peak velocity, anthropometrics, elite players, speed skating, ice hockey

## Abstract

There is no research examining female bandy players, which creates a gap of knowledge of female skating performance and its determinants with male skating performance, not only in bandy but also in exercise science in general. Therefore, the aim of this explorative study was to investigate position and playing-level differences in the sprint skating performance and anthropometrics of 74 elite female bandy players (age: 18.9 ± 4.1 years; height: 1.67 ± 0.06 m; body mass: 63.2 ± 7.4 kg). Participants were categorised according to playing level (26 elite and 48 junior elite players) and position (22 defenders, 35 midfielders, and 17 forwards). They were tested on their anthropometric characteristics and sprint linear skating profile over 80 m with the split times measured at 10, 20, 40, 50, 60, 70, and 80 m to calculate the average velocities between these different 10 m intervals. Results revealed that elite players had more training experience, were heavier, could accelerate faster, and reached a higher maximal velocity than the junior elite players (9.52 ± 0.37 vs 8.84 ± 0.40 m/s, respectively). In general, defenders were heavier than forwards, and the elite forwards accelerated faster in the first 10 m than the midfielders (*p* = 0.041). In summary, playing level and position, body mass, and training experience modulated skating sprint performance. The findings suggest that female junior-level players should spend more time developing skating sprint and acceleration abilities to meet the specific demands of playing at the elite level. Moreover, the coaches and professionals who work with female bandy players should be aware that the development of acceleration ability is more important for forwards.

## Introduction

Bandy is a team and ball sport often called winter football because it is played on a soccer-sized ice rink between two teams each consisting of 11 players, including a goalkeeper. All players use skates, and everyone but the goalkeeper has a club. The purpose of the game is to score goals by striking the ball into the opposing team´s net using the club within two halftimes of 45 min. It is a historical ancestor of ice hockey without clear distinction between the two sports until the 1920s. Bandy is the second most participated-in winter team sport after ice hockey, played both by men and women with over 350,000 practitioners (Persson et al., 2021). Moreover, women´s bandy has risen in popularity with the world championships taking place every second year.

However, despite the large number of bandy participants, there is no research examining female bandy players, creating a gap of knowledge of female physiology and performance, not only in bandy but also in sport in general. Unfortunately, this trend of women participants being significantly underrepresented in exercise science has been seen earlier across several influential sport-science journals (Costello et al., 2014; Mujika and Taipale, 2019). Thus, this significant research discrepancy usually leads to studies based on men being translated into the training and coaching practice with women, which is inadmissible when we know the performance differences between sexes in elite athletes across a variety of sports range between 8% and 10% (Sandbakk et al., 2018).

Everything we know about the nature of the bandy game is based on a few older (Häkkinen and Sinnemäki, 1990; Häkkinen and Sinnemäki, 1991) and recent studies (Blomqvist et al., 2018; Johansson et al., 2021; Persson et al., 2021; Petré et al., 2022) conducted with male players. Accordingly, we know that bandy is a high-intensity intermittent sport that requires players to repeatedly engage in sequences of intense skating activities (e.g., sprinting, changes of direction, acceleration, deceleration) on the bandy pitch (Blomqvist et al., 2018; Johansson et al., 2021; Persson et al., 2021). Therefore, it imposes high internal and external workloads on the players. For instance, elite male bandy players spend 40–80 min (i.e., 36%–71% of the playing time) in a heart rate zone between 81% and 100% of their maximum, with 15–27 min above their lactate threshold during a match, depending on the player’s position (Blomqvist et al., 2018).

Furthermore, recent studies have revealed that the total distance covered in bandy can vary between playing positions, with defending players covering more distance (23.2 ± 2.4 km) than offensive players (21.1 ± 3.5 km), but at a lower mean skating velocity (Blomqvist et al., 2018; Persson et al., 2021). These differences probably contribute to superior aerobic power (i.e., VO_2max_) in forwards compared to defenders (59.8 ± 4.3 and 53.0 ± 5.6 mL/kg/min, respectively) (Petré et al., 2022). In addition, elite players cover a distance of 450 m performing high- and very high-intensity accelerations and decelerations, skating ≈2.4 km very fast and ≈600 m sprinting during a match play (Johansson et al. (2021).

Thus, it is reasonable to assume that high-velocity skating, that sometimes reaches over 37 km/h is one of the most important skills in bandy as it is necessary to win ball possession or to outperform an opponent (Blomqvist et al., 2018; Johansson et al., 2021; Persson et al., 2021). For instance, in hockey, which has similarities to bandy concerning the skating movement pattern, acceleration ability and maximal velocity increase the probability of winning (Renaud et al., 2017; Douglas et al., 2019). Furthermore, it seems that performance requirements in bandy appear to be position-specific. The offensive players spend significantly more time in fast, very fast, and sprint skating than defensive players (Persson et al. (2021). Similar findings were reported by Douglas et al. (2019), who found that offensive players (e.g., forwards) in women´s world-class ice hockey spent more time in high-intensity skating and glided for a longer time compared to defensive players. Moreover, when testing in sprint skating, female hockey forwards were faster than players in the defensive positions (Wilson et al., 2021). Previous research, comparing playing levels in female ice hockey, revealed elite women’s ice hockey players were older and heavier, had less body fat, had higher aerobic and anaerobic capacity, were stronger and faster, and were involved in a higher number of movements at high intensity compared to younger and sub-elite counterparts during a match play (Bracko, 2001; Ransdell et al., 2013; Douglas et al., 2020).

Therefore, based on studies on ice hockey and due to the lack of studies on female bandy players, there is an evident need to start with descriptive investigation to understand the current level of skating sprint performance within elite and sub-elite female bandy players (Emmonds et al., 2019). Such information can be valuable to reveal if playing position and level potentially modulate sprint performance. Consequently, the aim of this study was to compare the anthropometrics and skating sprint profile of junior and senior elite female bandy players of the different positions. We hypothesised that the anthropometric and sprint skating profile would vary according to the different playing positions and playing level. Based on previous studies on bandy and hockey, it is reasonable to believe that offensive bandy players (forwards) and senior players will have higher skating velocity than other playing positions and junior players, respectively. Potential differences among playing positions and levels may provide better insight into the necessary sprint skating qualities important for success in that position and level. In addition, this information can be used to provide appropriately designed, both strength and conditioning, and technical training programmes for each playing position and playing level.

## Methods

### Participants

A group of 74 female bandy players (age: 18.9 ± 4.1 years; height: 1.67 ± 0.06 m; body mass: 63.2 ± 7.4 kg) participated in the study (Table 1). Players were categorised according to level (26 elite and 48 junior elite players) and playing position (22 defenders, 35 midfielders, and 17 forwards). No participants had reported a history of neuromuscular disease or injuries in the previous 6 months. Two days prior to the experimental visits, subjects were asked to avoid sleep deprivation, to refrain from high-intensity training, and to avoid tobacco, alcohol, and caffeine. Their training experience (years), weekly training frequency for different types of sessions (e.g., strength training, on- and off-ice training) during different periods (e.g., preparation and competition period) are presented in Table 1. All participants were fully informed verbally and in writing about the procedures and signed written consent (and by their parents when under 18 years of age) before participation. The study was conducted following the latest revision of the Declaration of Helsinki and current ethical regulations for research and was approved by the Swedish Ethical Review Authority (no. 2022–01550–01).

**TABLE 1:** Mean (±SD) anthropometrics and training experience for each playing position and level.

Parameter	Position					
	Elite			Junior elite		
Parameter	Defence	Midfield	Forward	Defence	Midfield	Forward
Number n)	9	14	3	13	21	14
Age (yrs)	24.3 ± 3.2^a^	23.2 ± 3.8	20.7 ± 1.2	15.9 ± 1.3^a^	16.1 ± 1.5^a^	17.6 ± 1.4
Height m)	1.70 ± 0.06	1.68 ± 0.05	1.69 ± 0.07	1.66 ± 0.05	1.66 ± 0.07	1.65 ± 0.04
Body mass (kg)	69.9 ± 9.8^a^	64.6 ± 6.2	65.0 ± 6.9	63.9 ± 7.3^a^	61.1 ± 7.3	59.5 ± 4.7
Playing experience (yrs)	16.9 ± 2.2^b^	15.9 ± 3.6^b^	11.0 ± 1.7	9.3 ± 0.9	9.3 ± 2.1	9.7 ± 3.4
*Preparation off-ice period (sessions per week)*						
Office	6.1 ± 0.5	5.6 ± 0.6	5.7 ± 0.6	5.5 ± 1.4	5.1 ± 2.2	4.8 ± 2.2
Strength training	2.4 ± 1.2	2.7 ± 1.6	2.0 ± 1.0	4.0 ± 2.2	3.3 ± 2.7	3.2 ± 2.5
*Preparation on-ice period*						
Off-ice sessions/week	5.6 ± 0.9	4.9 ± 0.6	5.0 ± 0.0	5.0 ± 0.9	5.5 ± 1.6	4.9 ± 1.7
On-ice sessions/week	3.1 ± 0.4	3.4 ± 0.6	3.3 ± 0.6	3.8 ± 1.4	4.3 ± 1.6	3.6 ± 1.4
Strength training	1.9 ± 1.0	1.6 ± 0.6	1.3 ± 0.6	2.0 ± 1.5	2.8 ± 1.8	2.2 ± 3.0
*Competition period*						
Off-ice sessions/week	5.9 ± 1.3	5.6 ± 0.6	4.3 ± 2.9	5.3 ± 1.1	3.9 ± 1.9	4.8 ± 2.3
On-ice sessions/week	4.0 ± 0.0	4.2 ± 0.4	3.0 ± 1.7	4.8 ± 1.2	3.3 ± 1.6	4.3 ± 2.2
Strength training	1.7 ± 1.1	1.4 ± 0.5	1.0 ± 1.0	2.1 ± 1.7	2.3 ± 1.8	1.8 ± 2.5

^a^ indicates a significant difference with forward players on a *p* < 0.05 level.

^b^ indicates a significant difference between the elite and junior groups for this position and with the forward position at the elite level on a *p* < 0.05 level.

### Procedure

All testing was carried out in the period between January and March 2022. All tests were conducted in an indoor facility to eliminate the effect of weather conditions on results. Before testing the sprint skating profile, anthropometric variables of height and body mass were measured in each subject. Thereafter, three participants at a time performed their own preferred warm-up for 15 min to be ready for testing 80-m maximal linear sprint skating. The participants wore their regular training gear during warm-up and testing. After their warm-up, the participants sat down on a chair and the testing procedure was described to them. After 5 min of rest, each participant performed two maximal 80-m sprint skating attempts with their club in their hand (to have the same condition as in competition), starting with the club behind the starting line.

Pairs of photocells (Egotest AS, Porsgrunn, Norway) were put at the start, 10, 20, 40, 50, 60, 70, and 80 m at 1.2 m height to measure times at these distances and to calculate the average velocities between these different distances. The participants started 0.5 m behind the first pair of photocells and had to skate for 90 m to avoid having to quit too early before the finish line. All participants were encouraged to skate as fast as possible over 90 m in a straight line. Each player repeated the same procedure for two attempts, and only the best time taken to cover 80-m distance in the sprint skating test was used in data analysis. A rest period of 5 min was provided between attempts.

### Statistical analysis

Data are expressed as mean ± SD. To compare the anthropometric, training experience, and sprint skating profile of the different playing positions and level, a 2 (level: junior and elite) x 3 (position: defenders, midfielders, and forwards) analysis of variance (ANOVA) was used. In addition, for the average velocity per 10 m interval, a 7 (average velocity per 10 m interval: repeated measures) x (level) x (position) ANOVA was used. Where significant differences were found, a Holm–Bonferroni probability adjustment *post hoc* test was used to determine the source(s) of those differences. Effect size was evaluated with η^2^_p_ (partial eta-squared), where 0.01< η^2^_p_<0.06 represents a small effect, 0.06< η^2^_p_<0.14 a medium effect, and η^2^_p_>0.14 a large effect (Cohen, 1988). All analyses were performed using SPSS Version 25.0. Statistical significance was set at *p* < 0.05.

## Results

No significant effect for playing positions and interaction (playing position*level) effects was found for height or sprint skating times (F ≤ 2.63, *p* ≥ 0.080, η^2^_p_ ≤ 0.07, Tables 1, 2). A significant effect of playing position for body mass (F = 3.5, *p* = 0.035, η^2^_p_ = 0.21) was found. Furthermore, for playing level a significant effect was found on body mass and sprint times from 10 m to 80 m (F ≥ 6.63, *p* ≤ 0.012, η^2^_p_≥0.09, Table 1) in which defenders were heavier than forwards, and elite players were heavier and faster at these distances than junior elite players. However, when analysed per position between elite and junior players, no significant differences in body mass were found (*p* ≥ 0.13). The elite players had significantly more training years of experience than the junior players (F = 17.2, *p* < 0.001, η^2^_p_ = 0.60, Table 1). In addition, a significant effect of position and interaction effect was found for years of experience and age (F ≥ 3.3, *p* ≤ 0.042, η^2^_p_≥0.11, Table 1). Post-hoc comparison revealed that the elite forward players had fewer years of training experiences and were younger than those in the other positions, while training experience was the same for the players at the junior level, but forwards were significantly older than the players in the other positions (Table 1). No other significant effects on training sessions per week during the different periods were found for level and positions (F ≤ 3, *p* ≥ 0.062, η^2^_p_≤0.06, Table 1).

**TABLE 2:** Mean (±SD) sprint times at the different distances for each playing position and level.

Parameter	Position					
	Elite			Junior elite		
	Defence	Midfield	Forward	Defence	Midfield	Forward
Number n)	9	14	3	13	21	14
*Sprint times* *^a^*						
10 m s)	2.09 ± 0.11	2.09 ± 0.10	2.02 ± 0.03	2.22 ± 0.11	2.18 ± 0.10	2.13 ± 0.17
20 m s)	3.52 ± 0.18	3.50 ± 0.13	3.39 ± 0.06	3.73 ± 0.15	3.68 ± 0.15	3.61 ± 0.22
40 m s)	5.96 ± 0.28	5.93 ± 0.16	5.72 ± 0.11	6.32 ± 0.20	6.26 ± 0.25	6.16 ± 0.31
50 m s)	7.10 ± 0.33	7.06 ± 0.18	6.81 ± 0.13	7.52 ± 0.23	7.46 ± 0.30	7.34 ± 0.35
60 m s)	8.18 ± 0.37	8.15 ± 0.20	7.85 ± 0.17	8.68 ± 0.24	8.63 ± 0.35	8.48 ± 0.39
70 m s)	9.25 ± 0.42	9.21 ± 0.21	8.87 ± 0.21	9.83 ± 0.26	9.78 ± 0.41	9.61 ± 0.43
80 m s)	10.31 ± 0.48	10.27 ± 0.24	9.88 ± 0.25	10.97 ± 0.28	10.92 ± 0.47	10.73 ± 0.47

^a^ indicates a significant difference between the elite and junior groups for this position at each time on a *p* < 0.05 level.

**FIGURE 1:**
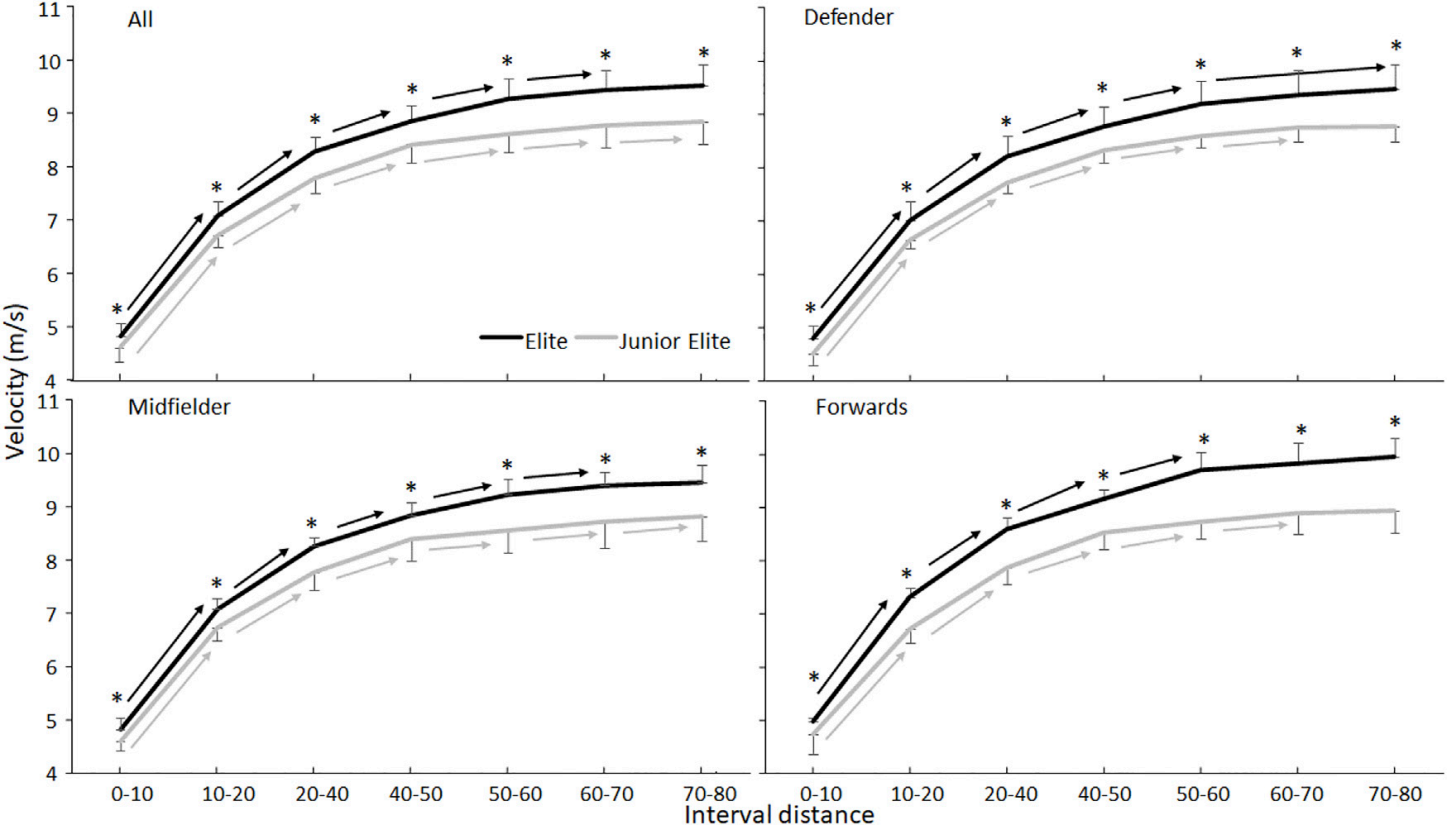
Mean velocity (SD) over 10 m distances averaged for all elite and junior players and per position in elite and junior players. * indicates a significant difference between elite and junior players at this interval distance on a *p* < 0.05 level. → indicates a significant increase in velocity between these two interval distances and all right of the arrow for this group on a *p* < 0.05 level.

When analysing the velocity over the 10 m intervals between level and position, a significant effect of velocity (F = 4,472, *p* < 0.001, η^2^_p_ = 0.99), level (F = 51.4, *p* < 0.001, η^2^_p_ = 0.43), and velocity*level interaction (F = 16.4, *p* < 0.001, η^2^_p_ = 0.19) and a near significant effect of position (F = 2.9, *p* = 0.058, η^2^_p_ = 0.08) were found. No significant effects were found for other interaction effects (F ≤ 0.91, *p* ≥ 0.536, η^2^_p_≤0.03). Post-hoc comparison showed that the elite players accelerated faster each 10 m and reached a higher maximal velocity (9.52 ± 0.37 vs 8.84 ± 0.40 m/s) (Figure 1). When evaluating per position between levels, all elite players in all positions accelerated faster each 10 m. Maximal velocity was reached for 70% of the elite players between 70 and 80 m, while 62% of the juniors reached their maximal skating velocity in this segment. The elite forwards accelerated faster in the first 10 m than the midfielders (*p* = 0.041), thereby causing a near significant position effect (Figure 1).

## Discussion

This study is the first to investigate the linear sprint skating performance and anthropometrics of elite senior and junior female bandy players with a specific focus on playing positions. The main findings were that elite players were heavier, could accelerate faster, and reached a higher maximal velocity than junior elite players. Moreover, defenders were heavier than forwards, and elite forwards accelerated faster in the first 10 m than midfielders.

It is evident that women’s bandy has been underrepresented in sports science, without a single performance profiling study being published. Therefore, it is impossible to compare the anthropometrics and skating performance data from the current study to any previous research. However, the results can be compared to the findings of previous studies that investigated only elite male bandy players (Blomqvist et al., 2018; Johansson et al., 2021; Persson et al., 2021; Petré et al., 2022). In general, both senior and junior female players from the current study were lighter and shorter (63.2 ± 7.4 kg and 1.67 ± 0.06 m) than the elite male bandy players from previous studies (82.8 ± 4.2 kg and 1.81 ± 0.04 m (Johansson et al., 2021; Persson et al., 2021); (83.8 ± 7.6 and 182.4 ± 6.6; (Petré et al., 2022). These differences can be attributed to well-documented sex differences in general, with the average man being taller (≈13 cm), heavier (14–18 kg), and with more muscle mass (18–22 kg) and less fat mass (three to six kg) than the average woman (Kenney et al., 2021). Likewise, male ice hockey players are taller (≈12–14 cm) and heavier (≈8–10 kg) than female players (Gilenstam et al., 2011; Bigg et al., 2021; Czeck et al., 2022).

Because of similarities (skating patterns) between ice hockey and bandy, we think that it is reasonable to compare female players in these two sports. In general, female bandy players are lighter (≈5–10 kg) and slightly shorter (≈two to four cm) compared to female hockey players (Bracko and George, 2001; Ransdell et al., 2013; Douglas et al., 2019; Douglas et al., 2020; Bigg et al., 2021; Czeck et al., 2022). The differences are more exaggerated when our cohort is compared to elite players and subjects from recent ice hockey studies (Douglas et al., 2019; Douglas et al., 2020), which can be explained by several facts that differentiate the sports. Firstly, even though ice hockey rules for women do not allow body checks, it is still a game full of contact compared to bandy. Therefore, heavier and stronger bodies can provide an advantage during collisions between ice hockey players moving at high speeds. Secondly, lighter bodies could be more energy economical for bandy players, who effectively play between 70 and 90 min, compared to hockey players, who are effectively engaged for 15–25 min of multiple sprint bouts (Lignell et al., 2018; Persson et al., 2021). Furthermore, despite these dissimilarities between the sports, in both earlier (Bracko, 2001; Bracko and George, 2001; Ransdell et al., 2013) and recent studies (Douglas et al., 2019; Douglas et al., 2020), elite women players were heavier than sub-elite players, which is in line with our findings. Even though we did not examine lean body mass and fat percentage, the differences in body weight might partially explain differences in skating sprint performance between playing levels.

In the current study, all elite players in all positions accelerated faster each 10 m, reaching a higher maximal velocity over 80 m than the junior players. Moreover, 70% of the elite and 62% of the junior players reached maximal velocity between 70 and 80 m. This is in line with other studies demonstrating that elite female (Bracko and George, 2001) and elite male hockey players are faster than sub-elite players (Buckeridge et al., 2015; Peterson et al., 2015; Renaud et al., 2017; Vigh-Larsen et al., 2019). Moreover, Douglas et al. (2020) showed that elite female hockey players are involved in higher numbers of on-ice explosive efforts and high-intensity skating in both absolute and relative terms (per minute) than sub-elite players, which could be similar to bandy. Although elite women players were heavier than junior players in the present study, it is reasonable to assume that this extra body mass is caused by more lean body mass and less fat%. This enabled them to generate more relative power, which is in line with studies by Vigh-Larsen et al. (2019); Ransdell et al. (2013), who found that in men’s and women’s hockey, elite players, despite being heavier, also had a higher skeletal muscle mass and lower body fat percentage compared to sub-elite and junior players. This could inherently enable them to produce more force, accelerate faster and reach higher maximal velocity than junior players. Similarly; Gilenstam et al. (2011) reported that female hockey players with higher lean body mass and lower fat percentage have better skating acceleration and speed performance. Moreover, it seems that elite ice hockey players, irrespective of sex, are stronger and have better jump and sprint performance than lower-level players (Peterson et al., 2015; Shell et al., 2017; Vigh-Larsen et al., 2019; Budarick et al., 2020), which might apply in bandy players.

In addition, several studies have reported that faster skating performance of elite hockey players can be explained by better skating technique (e.g., larger range of motion and stride width, greater stride rate and length), which enables them to optimally apply force in the push-off for propulsion and effective acceleration (Upjohn et al., 2008; Stull et al., 2011; Renaud et al., 2017; Shell et al., 2017). Given that elite bandy players from the current study had significantly more training experience (≈5–7 years), it is reasonable to conclude that longer playing time and involvement in systematic bandy training could lead to bigger accumulated training effects and advanced skating performance. In brief, it seems that a greater amount of learning time (i.e., the law of practice) (Schmidt et al., 2018) through organised bandy training might enable elite players to learn and store more sport-specific programmes (Pojskic et al., 2018; Pojskic et al., 2019), which could, in turn, facilitate advanced acquisition of skating skills compared to junior players. In support of this, based on previous training studies, it could be seen that systematically designed off- and on-ice strength and conditioning training led to improvement in skating acceleration and speed (Lee et al., 2014; Dæhlin et al., 2016), with superior training effects observed when skating-specific resistance-based exercises (e.g., bungee skate training) were used (Janot et al., 2013).

Similarly to other team sports (e.g., football, basketball, hockey), playing positions in bandy are well defined (i.e., defenders, forwards, midfielders), with players being involved in different tasks (e.g., stopping the opponents, connecting defence and offense, scoring) that are highly specific to different playing positions (Persson et al., 2021). Therefore, it was logical to believe that the measured anthropometrics and skating performance would have power to discriminate between the playing positions. Namely, in the present study, defenders were heavier than forwards across the whole cohort, and the elite forwards accelerated faster in the first 10 m than the midfielders. Due to the lack of women’s bandy studies, direct comparisons of anthropometrics and skate performance across playing positions between studies cannot be made. Moreover, there is only one study recently published that investigated physical characteristics (e.g., body mass and height, aerobic power, lower-body power and strength) of elite male bandy players across playing positions. In brief, Petré et al. (2022) found only superior VO_2max_ in forwards compared to defenders, while there were no significant differences for any other measurements between playing positions. However, as in our sample of women, defenders in their study were heavier than forwards, even though the difference was not significant (86.5 ± 8.3 kg vs 83.1 ± 7.8 kg, respectively). Similarly, in two recent female hockey studies, defenders were heavier than forwards, but with differences being more exaggerated at lower playing levels (Douglas et al., 2020; Czeck et al., 2022). Given that forwards’ main task is to attack and score by quick skating into the penalty area where they receive and strike the ball, it is logical to assume that they would be the fastest players on the ice rink. Having lower body mass can potentially provide them greater relative strength and power, which inherently could enable them to efficiently overcome their body inertia and consequently accelerate at a faster rate (Sekulic et al., 2021).

Considering the recent motion analyses in male bandy, we assumed that offensive players, due to the specific game roles, would be faster in the sprint skating measurements. In brief, the offensive players (i.e., forwards) spent more time in the fastest velocity zones (i.e., 20–25, 25–30, and >30 km/h) than defensive players (i.e., libero and halves) (Blomqvist et al., 2018; Persson et al., 2021), which is the pattern seen during ice hockey matches too (Jackson et al., 2017; Douglas et al., 2019). It seems that the offensive players’ roles, which involve more time in fast velocity zones and high-intensity activities during the match play (Blomqvist et al., 2018; Persson et al., 2021), contributed to the advanced acceleration ability in offensive players compared to midfielders. While midfielders are usually most skilled and proficient in bandy ball handling technique and capable of winning the ball from the opposition and passing it to the forwards, the latter must be able to accelerate very fast to fulfil their position-specific tasks efficiently. Interestingly, even though elite forwards had less playing experience than other positions, they were faster skaters. It seems that time spent playing in a specific playing position is more important than overall playing experience when it comes to specific adaptation and advanced skating performance. However, a difference in skating print performance was only seen in elite senior women players. Apparently, there was no clear differentiation between playing positions in skating performance in junior players, which can be attributed to their less pronounced physical maturity (Bracko and George, 2001). In addition, in view of the juniors’ age (16.5 ± 1.4 years), we may assume that they have not been involved in position-specific training programmes to date compared to senior players. Furthermore, neither Petré et al. (2022) nor Czeck et al. (2022) found differences between defenders and forwards in the skating sprint measurements in male bandy players and female hockey players, respectively. However, these authors measured skating speed over 15 and 30 m, as well as 30-m flying speed, which makes comparisons between our studies difficult. It seems these inconsistent findings might be dependent on several factors, such as competition sex, playing level, and applied measurements, which specifically impose needs for further studies on women’s bandy.

The strength of the present study is that this is the first study that investigated the anthropometric and skating sprint profile in female bandy players compared to previous older (Häkkinen and Sinnemäki, 1990; Häkkinen and Sinnemäki, 1991) and recent studies (Blomqvist et al., 2018; Johansson et al., 2021; Persson et al., 2021) that only included male players. Moreover, this is the first study that successfully implemented the accurate on-ice measurement protocols to assess the acceleration and maximal velocity profile in elite female bandy players in long-distance sprint skating (10–80 m), which increases the ecological validity of the study. Even though the findings from this study unequivocally add to the pool of research knowledge on women’s bandy, there are several limitations that should be acknowledged. Firstly, there is a risk that a smaller number of elite players and an unequal number of players in different playing positions could underestimate the power of the analyses and provide less valid data. For instance, there were only three forwards at the elite level, which might decrease their comparability with other playing positions. Secondly, to provide better body-build indices, the lean body mass and fat percentage should have been measured. To explain skating differences between playing levels and positions, analyses of skating technique and physical capacities are warranted.

## Conclusion and practical applications

Body mass, playing level, position, and training experience modulated skating sprint performance in female bandy players. Elite senior bandy players proved to be heavier and superior in skating sprint performance compared to their elite junior peers. It seems that advanced skating performance in the senior elite group may have been facilitated by both the repetition of the bandy-specific actions and the amount of learning time. The findings suggest that female junior-level players should spend more time developing skating sprint and acceleration abilities to meet the specific demands of playing at the elite level. However, we should not ignore the fact that the elite cohort was the product of a long-term selection process. Moreover, the coaches and professionals who work with female bandy players should be aware that the development of acceleration ability is more important for offensive players (e.g., forwards). Given that playing position modulated the acceleration ability only in senior players, the development of position-specific training would have greater value for junior players who aim to compete at the senior level.

## Data Availability

The raw data supporting the conclusion of this article will be made available by the authors, without undue reservation.
